# Participatory research and community engagement in climate and health research

**DOI:** 10.2471/BLT.25.294164

**Published:** 2026-03-01

**Authors:** Yasna Palmeiro-Silva, Admire Nyamwanza, Anh Ngoc Vu

**Affiliations:** a Center for Health and the Global Environment, University of Washington, Hans Rosling Center, 3980 15th Ave NE, Seattle, Washington 98105, United States of America.; b Institute for Global Health and Development, Queen Margaret University, Edinburgh, Scotland.; c National Centre for Social Research, London, England.; Correspondence to Yasna Palmeiro-Silva (email: palmeiro@uw.edu).

Human-induced climate change is one of the defining health challenges of the twenty-first century. The World Health Organization estimated in 2014 that climate change will be linked to approximately 250 000 additional deaths per year between 2030 and 2050.^1^ These deaths, caused by undernutrition, malaria, diarrhoea and heat stress, will disproportionately affect the most vulnerable populations.^2^^,^^3^ Therefore, generating evidence on the interactions between climate change and health is a priority for planning effective adaptation and mitigation strategies^2^ across multiple governance levels.

Designing and conducting such research carries ethical questions, especially amid climate and sociopolitical uncertainties, and methodological limitations.^4^ Research often involves communities that are already socioeconomically or politically marginalized, and whose capacity to influence decision-making is limited. In this scenario, participatory research^5^^,^^6^ and community engagement^7^^,^^8^ are often advocated as best practices. Yet their implementation presents practical and ethical challenges, including questions such as who defines research agendas; what constitutes meaningful participation and why it should be pursued; and how and why diverse forms of knowledge and perspective are negotiated in research design, analysis and dissemination.

Additionally, research on climate change and health presents ethical considerations that extend beyond standard participatory frameworks. These concerns include the compounding nature of climate-related threats to livelihoods that worsen existing vulnerabilities; the urgency of adaptation interventions, which often conflict with the time participatory processes require; and the governance implications of research findings, which inform policy in a complex landscape spanning local, national and international levels.

Here we present lessons from two case studies that were discussed at the 2024 Global Forum on Bioethics in Research under Theme 1: interdisciplinary approaches, co-production and integrating Indigenous knowledge.^9^ Based on these cases, we argue that ethical participatory research on the health impacts of climate change, adaptation and mitigation (among others), requires a shift towards ongoing negotiation of power among all participants and stakeholders, participatory establishment of priorities and holistic risk assessments. These processes demand flexibility, reflexivity and genuine commitment to community participation and well-being.

## Case studies 

An impact assessment implemented between 2019 and 2022 evaluated the nutritional and psychosocial health outcomes of three climate adaptation actions in rural mid-Zambezi Valley, Zimbabwe, a recognized climate hotspot prone to recurrent droughts.^10^ The study reflects on three ethical issues that arose during the assessment. 

First, researchers should guarantee that the selection of adaptation actions whose health outcomes are to be evaluated is conducted with explicit participation of and in consultation with the affected communities. Researchers should also ensure the involvement of the most vulnerable in targeted communities, such as women and resource-constrained households, in line with justice and fairness in research. This approach, analysed from the epistemic justice angle (that is, valuing the knowledge and expertise of diverse individuals and communities), promoted community participation throughout the research process, from the identification of relevant adaptation actions to refining research questions, while valuing local knowledge. Doing so was essential because the study sought to reach meaningful recommendations to address the various challenges that arise during the evaluated actions and the decisions that need to be made. 

Second, the complexity of climate change requires diverse perspectives and methodological approaches, primarily to ensure that the right research questions are prioritized in engaging communities, which is sometimes challenging due to power dynamics. In this case, the project was convened as multidisciplinary, therefore promoting equal respect among researchers, participants and their communities. 

Finally, the obligations that researchers have towards research participants exposed to dangerous climate risks were clearly stated. This ethical issue arose in the context of the case study site being located in an area exposed to the effects of increasing climate change-related drought cycles. Researchers included activities to manage expectations and increase transparency and communication; they also provided post-research support.

The second case study analysed the health risks and adaptive capacities of informal outdoor workers in Viet Nam facing climate hazards such as heatwaves and extreme rainfall.^11^ The study identified three ethical challenges. First, the hidden risk that empowerment and consciousness-raising through co-creation might expose workers to reprisals from employers or the State, given their informal and precarious status. Second, the necessity of acknowledging that climate change exacerbates pre-existing intersecting vulnerabilities, such as job insecurity and social exclusion, avoiding the overshadowing of these immediate concerns. Third, the difficulty in translating research findings into effective public policies benefiting these workers. The project sought to mitigate these risks through ensuring confidentiality; adapted informed consent processes; integrating climate resilience with socioeconomic improvements; and close interdisciplinary collaboration with local partners to ensure policy relevance and applicability.

## Ethical dilemmas 

The intersection of climate change, climate and health research presents various ethical dilemmas in research prioritization, particularly when employing participatory approaches with vulnerable communities. The case studies from Viet Nam and Zimbabwe reveal three main ethical dilemmas.

First, the dilemma of whose priorities shape the research agenda emerges when the scientific objectives of external researchers diverge from the communities’ immediate needs. This dilemma was observed in Zimbabwe, where researchers initially selected climate adaptation actions they thought were critical for evaluation but that were not a local priority. Although researchers had initially identified specific climate adaptation interventions, they changed them after considering communities’ expressed concerns about psychosocial distress from droughts. Here, community members’ experiential knowledge of drought patterns directly shaped the selection of adaptation interventions to be evaluated. Consultations happened through iterative processes, expanding the research scope and health outcomes, and demonstrating how epistemic justice involves genuine negotiation rather than simple one-way consultation. Ordinary participatory research focuses on such generic ethics principles as inclusion and informed consent. However, this dilemma highlights the challenges of adjusting the scope of research and ensuring that both community knowledge and technical expertise are considered in climate and public health work.

Second, balancing scientific rigour with participatory integrity presents ongoing challenges, as reflected in the Zimbabwe study regarding community involvement in selecting relevant adaptation actions for evaluation. Traditional epidemiological methods usually follow standardized protocols that might constrain community input and participation, while fully participatory approaches might compromise overall generalizability, as what would work for one community might not work for another. The Zimbabwe study addressed this challenge through a community-led co-identification of relevant adaptation strategies. This approach recognized that methodological pluralism (that is, valuing diverse ways of knowing) enhances research quality, and that co-production with the community is desirable. This approach means that in climate–health research, using multiple methods should go beyond a narrow focus on inclusion, and be understood as an ethical obligation to improve data quality and support beneficial outcomes. The implication in the Zimbabwe case is that had the community not been involved, researchers would have evaluated adaptation actions that were unimportant to the community, leading to locally irrelevant results.

Third, the temporality dilemma arises when urgent climate actions and policies conflict with the time required for meaningful participation. For example, in the Viet Nam case, striking a balance between a just and inclusive research process and research and policy output needs was required. Fast research timelines driven by systems evaluations, publications and funding cycles or policy windows may undermine the relationship building that is essential for ethical engagement. In Viet Nam, pressures to quickly assess heat exposure impacts conflicted with building trust with informal workers, a process that sometimes required extended engagement periods. Researchers resolved this dilemma by assigning flexible funding arrangements, and adopting phased approaches where preliminary findings could inform policy while deeper participatory processes continued. This case illustrates how funders, mandating institutions and ethics committees can recognize and accommodate staged participatory processes in climate–health research, allowing iterative engagement phases that can adapt to community needs, evolving climate and weather conditions and timely research findings.

These dilemmas reveal that ethical participatory research in climate change and health extends beyond procedural compliance to encompass transformative engagement. This transformation involves: (i) recognizing communities as knowledge producers rather than data sources, with their experiential understanding of climate impacts holding equal validity to scientific measurements; (ii) acknowledging that vulnerability to climate change intersects with existing structural inequities, requiring research designs that address rather than reproduce these power asymmetries; and (iii) ensuring that research outcomes translate into timely and tangible benefits for participants, whether through direct support services, capacity-building or policy advocacy.

The journey from ethical reflections to on-the-ground participatory research is not a linear exercise but a continuous cycle of reflection, action and learning. Participatory approaches in climate and health research must navigate between respecting community autonomy and fulfilling scientific obligations; between urgent action and deliberative process; and between local specificity and broader applicability. Success lies in transparently negotiating tensions and dilemmas through ongoing dialogue, reflexivity and adaptive governance structures that privilege community well-being while generating robust evidence for climate action.
